# El Hierro Genome Study: A Genomic and Health Study in an Isolated Canary Island Population

**DOI:** 10.3390/jpm14060626

**Published:** 2024-06-12

**Authors:** Marta Puga, Javier G. Serrano, Elsa L. García, Mario A. González Carracedo, Rubén Jiménez-Canino, María Pino-Yanes, Robert Karlsson, Patrick F. Sullivan, Rosa Fregel

**Affiliations:** 1 Genomics and Health Group, Department of Biochemistry, Microbiology, Cell Biology and Genetics, Universidad de La Laguna (ULL), 38200 La Laguna, Spain; martagpuga@gmail.com (M.P.); elsa.gabi.lgg@gmail.com (E.L.G.); mgonzalc@ull.edu.es (M.A.G.C.); mdelpino@ull.edu.es (M.P.-Y.); 2 Evolution, Paleogenomics and Population Genetics Group, Department of Biochemistry, Microbiology, Cell Biology and Genetics, Universidad de La Laguna (ULL), 38200 La Laguna, Spain; jgonzase@ull.edu.es; 3 Genetics Laboratory, Institute of Tropical Diseases and Public Health of the Canary Islands (IUETSPC), Universidad de La Laguna (ULL), 38200 La Laguna, Spain; 4 Genomics Service, Servicio General de Apoyo a la Investigación, Universidad de La Laguna (ULL), 38200 La Laguna, Spain; rjimenec@ull.edu.es; 5 CIBER de Enfermedades Respiratorias, Instituto de Salud Carlos III, 28029 Madrid, Spain; 6 Instituto de Tecnologías Biomédicas (ITB), Universidad de La Laguna (ULL), 38200 La Laguna, Spain; 7 Department of Medical Epidemiology and Biostatistics, Karolinska Institutet, 171 77 Stockholm, Sweden; robert.karlsson@ki.se (R.K.); patrick.sullivan@ki.se (P.F.S.); 8 Departments of Genetics and Psychiatry, University of North Carolina, Chapel Hill, NC 27599, USA

**Keywords:** Canary Islands, isolation, precision medicine

## Abstract

El Hierro is the smallest and westernmost island of the Canary Islands, whose population derives from an admixture of different ancestral components and that has been subjected to genetic isolation. We established the “El Hierro Genome Study” to characterize the health status and the genetic composition of ~10% of the current population of the island, accounting for a total of 1054 participants. Detailed demographic and clinical data and a blood sample for DNA extraction were obtained from each participant. Genomic genotyping was performed with the Global Screening Array (Illumina). The genetic composition of El Hierro was analyzed in a subset of 416 unrelated individuals by characterizing the mitochondrial DNA (mtDNA) and Y-chromosome haplogroups and performing principal component analyses (PCAs). In order to explore signatures of isolation, runs of homozygosity (ROHs) were also estimated. Among the participants, high blood pressure, hypercholesterolemia, and diabetes were the most prevalent conditions. The most common mtDNA haplogroups observed were of North African indigenous origin, while the Y-chromosome ones were mainly European. The PCA showed that the El Hierro population clusters near 1000 Genomes’ European population but with a shift toward African populations. Moreover, the ROH analysis revealed some individuals with an important portion of their genomes with ROHs exceeding 400 Mb. Overall, these results confirmed that the “El Hierro Genome” cohort offers an opportunity to study the genetic basis of several diseases in an unexplored isolated population.

## 1. Introduction

**Figure 1:**
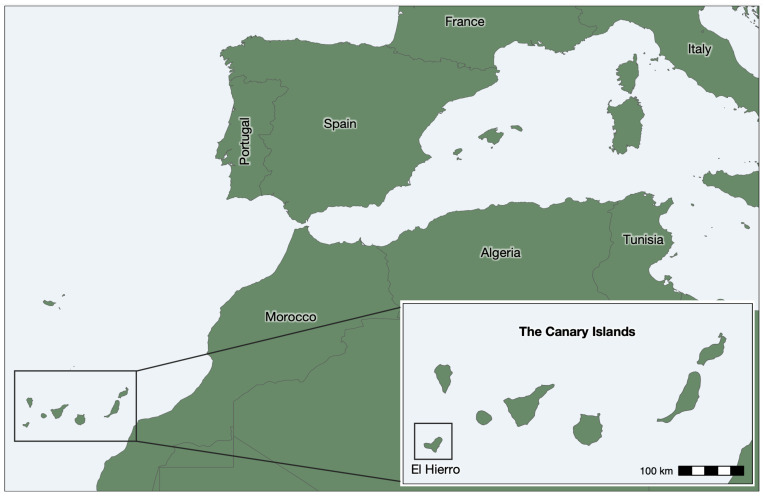
Map showing the location of El Hierro.

The Canary Islands are an archipelago of eight volcanic islands located in the Atlantic Ocean, 100 km west off the coast of Morocco (Figure 1). These islands are the outermost region of Spain and represent an autonomous community divided into two different provinces: Santa Cruz de Tenerife in the west (El Hierro, La Palma, La Gomera, and Tenerife) and Las Palmas in the east (Gran Canaria, Lanzarote, La Graciosa, and Fuerteventura). Each island has different characteristics in terms of their history, climate, and physical geography, potentially affecting the genetic composition and health status of its current inhabitants. 

Several genetic studies have been carried out on the Canarian population, both in ancient and modern populations [1,2,3,4,5,6,7,8,9]. These studies have shown that the demographic history of its population is complex due to the migration of people from several geographical regions. Genetic research—along with anthropological, archaeological, and cultural evidence—indicate that the most likely origin of the first Canarian inhabitants was North Africa [9,10]. Radiocarbon dating points to a first settlement at the beginning of the first millennium AD [11], while other studies suggest that different migration waves could have reached different islands [12]. The islands were rediscovered by European navigators in the 13th century, and from the 14th century onward, their geostrategic value increased as a stopping point for expeditions to the Americas [13]. In the 15th century, the Spanish conquest took place and although some of the settlements were initially peaceful, the revolts against the European occupation and the trading of enslaved natives led to significant mortality and forcible relocation of the indigenous population. After the conquest, the surviving indigenous people mixed with the European colonizers, mainly from the Iberian Peninsula, but also with Africans and Amerindians [14]. Enslaved people from North and sub-Saharan Africa were brought to the islands as workforce [15], while people from the Americas came due to the importance of the islands for the trade with these continents [16]. 

In this way, this complex, multi-ancestral background forms the basis of the current Canarian population. Several studies have explored how admixture has shaped the genetic composition of the Canary Islands, including through analyses of mitochondrial DNA (mtDNA), the Y chromosome, selected autosomal loci and, more recently, genome-wide data [17,18,19,20,21,22,23,24]. Results obtained for the present-day population compared with the indigenous people show the impact of European colonization. However, in all cases, a North African component has been identified that can be linked to the contribution of the Canarian natives. Admixture estimates range from 50% for the maternal mtDNA inheritance to less than 10% when considering the paternal lineages, with autosomal data pointing to an intermediate value of ~20% [12].

In recent decades, genetic research has made progress in understanding the genetic variation in individuals and populations in the identification of human traits and diseases. Following achievements in the study of monogenic diseases, the focus is now on common and complex diseases such as cancer, cardiovascular disease, diabetes, or psychiatric disorders, which are of an unquestionable interest for public health [25,26]. Complex diseases are characterized by polygenicity, with the involvement of many different loci, along with the participation of environmental factors [26]. Numerous associations between complex diseases and loci have been published [27]. 

Some approaches to the study of complex diseases focus on genetically isolated populations given their potential advantages [28] due to the small numbers of founders, geographic or cultural isolation, and population expansion, resulting in lower genetic diversity. These populations are characterized by reduced haplotype complexity since linkage disequilibrium (LD) patterns tend to extend over longer distances. In addition, certain alleles can persist over time or become extinct due to genetic drift, giving rise to a higher/lower prevalence of certain diseases compared with other populations. Therefore, the advantage of studies of isolated populations translates into simplifying the search process for any genetically determined trait. In general, these advantages have been exploited in diseases caused by rare variants [29], but there are currently numerous studies that use these isolated populations to clarify the underlying etiology of complex and common diseases [30]. 

Genetic isolation has already been described for the indigenous people of the Canary Islands (particularly for the smaller islands of La Gomera and El Hierro) [4,5,9] but also for the present-day populations of these islands [23]. El Hierro is the smallest of the main islands, with a current population of 11,646 [31], and it is the furthest from the African continent (Figure 1). Based on paleogenomic data, it has been proposed that the indigenous colonization of this island involved just one migration wave [5] and that its inhabitants, known as Bimbapes, remained isolated afterward [9]. After the European conquest, there was greater population movement between the islands, although this was less pronounced in El Hierro [32]. Throughout the 20th century, the economic and social development of El Hierro was slower than in other regions of the Canary Islands due to its limited farmland, inconsistent water supplies, and greater geographical distance. Many inhabitants were forced to emigrate, while immigration was minimal. In this way, the isolation and reduced population size likely resulted in a greater consanguinity and genetic drift trend than those observed in the other islands [5,23]. 

Nowadays, with the ease of mobility, there are few regions around the world where the characteristics of population isolation persist. El Hierro has been postulated as a genetic isolate based on genetic data from small samples [12,23]. Here, we introduce the El Hierro Genome study, a dataset containing genomic, sociodemographic, and health-related information from 1054 individuals from El Hierro. Our goals were the following: (1) evaluate the health status of the El Hierro population; (2) understand the genetic admixture in the island of El Hierro due to indigenous and colonizing peoples; (3) assess the signature that isolation has left in their genomic makeup.

## 2. Methods

This study was reviewed and approved by the Complejo Hospitalario Universitario de Canarias Ethical Committee (approval CHUNSC_2021_04), and all participants provided written informed consent for participation. 

Subjects. Recruitment was carried out from March 2021 to December 2021 in the Hospital Insular Nuestra Señora de los Reyes (HINSR), the only hospital in El Hierro. We invited adults (≥18 years old) with at least their maternal or paternal line (two grandparents from the same line) from El Hierro to participate. A blood sample was obtained from each participant at the HINSR clinical laboratory as part of a clinical blood test required for a general health check or because the participant had been invited to participate in the study. Each participant was interviewed to complete a demographic and health questionnaire recording their personal and family history of different diseases. The questionnaires were complemented with hospital medical record data. 

Genome-wide genotyping. DNA purification was carried out at the Servicio de Genómica, Servicios Generales de Apoyo a la Investigación, University of La Laguna (SEGAI-ULL). DNA was extracted from blood samples with the Mag-Bind HDQ Blood DNA & Tissue 96kit (Omega Bio-Tek, Norcross, GA, USA) using a Microlab^®^ STAR Line Starlet Replicator 96/2 (Hamilton). Genome-wide genotyping was performed with the Illumina Global Screening array (v3) at SciLifeLab (Uppsala, Sweden, https://www.scilifelab.se, accessed on 25 April 2024). Genotype calling was conducted using GenomeStudio (v2.0.3) (Illumina, San Diego, CA, USA). Quality control (QC) procedures were performed using PLINK (v1.9, v2) [33,34] and R [35]. SNPs were removed for call rates < 0.99, a minor allele frequency < 0.01, or extreme deviations from Hardy–Weinberg equilibrium (*p* < 1 × 10^−6^). Nine individuals were removed because they had genotype missingness > 0.01 or disagreement between their phenotype and their genetic sex. Following QC, 1045 subjects and 441,692 autosomal SNPs were retained.

Population genetic analysis. For this analysis, we only included (a) subjects whose four grandparents were born in El Hierro, (b) individuals without close family relatedness, and (c) subjects without extreme deviations in their ancestral composition. Briefly, we calculated π values between pairs of individuals using the --genome option from PLINK (v1.9). Then, because of the high consanguinity that is expected in El Hierro, we removed one of the subjects from pairs with π > 0.1875, which is the halfway point between second- and third-degree relatives. Two additional individuals were removed as they were identified as principal component analysis (PCA) outliers (see below). After this QC step, a total of 416 subjects (219 females and 197 males) were included in these analyses. MtDNA and Y-chromosome haplogroups were obtained using Haplogrep 2 [36] and SNAPPY [37], respectively. PCAs and run of homozygosity (ROH) inferences were performed using PLINK (v1.9). For the PCA, the El Hierro Genome dataset was merged with reference populations in order to understand the genetic ancestry of this population. First, El Hierro data were merged with data from the 1000 Genomes Project [38], and only common SNPs were retained (allele frequency > 0.01). This dataset included 2899 individuals and 244,496 SNPs. Variants in LD were removed using PLINK (--indep-pairwise 200 25 0.4), retaining a total of 187,651 SNPs. 

Second, as no North African individuals were genotyped in the 1000 Genomes project, we prepared a second dataset including samples from North Africa, sub-Saharan Africa, the Middle East, and Europe. We used data from Henn et al. [39] (18 individuals from North Morocco, 16 from South Morocco, 18 from Western Sahara, 19 from Algeria, 18 from Tunisia, 17 from Libya, 19 from Egypt, and 20 individuals from the Basque Country in Spain), Botigué et al. [22] (17 individuals from Galicia, 17 from Andalusia, and 17 from the Canary Islands in Spain), Arauna et al. [40] (28 individuals from Morocco, 20 from Algeria, 17 from Tunisia, and 19 from Syria), and Guillén-Guío et al. [23] (429 individuals from the Canary Islands), as well as European and sub-Saharan African populations from the 1000 Genomes Project. After removing related individuals, as explained above, 2091 individuals were included. However, the overlap between datasets was poor, producing only 19,753 SNPs. For that reason, each dataset was individually imputed using the TOPMed Imputation Server [41] and the TOPMed r3 reference panel. Imputed data were filtered to retain variants with *r*^2^ > 0.8 using bcftools (v1.9) [42]. Then, data from the different datasets were merged, and imputed variants with a genotype probability higher than 0.9, a genotyping rate of 0.99, and a minimum allele frequency of 0.01 were retained using PLINK (v1.9). Individuals with a genotyping rate lower than 90% were discarded. After removing variants in strong LD, the final dataset consisted of 1996 individuals and 225,866 SNPs.

ROHs were estimated following Ceballos et al. [43]. Briefly, we used PLINK with the following specifications: --homozyg-snp 50 --homozyg-kb 300 --homozyg-density 50 --homozyg-gap 1000 --homozyg-window-snp 50 --homozyg-window-het 1 --homozyg-window-missing 5 and --homozyg-window-threshold 0.05. 

Comparison with ancient Bimbape individuals of El Hierro. To obtain a comprehensive understanding of ancestry and isolation throughout the history of the island, we included in the analyses four indigenous individuals from El Hierro (13th–14th century Bimbapes; from Serrano et al. [9]). Firstly, we converted the imputed dataset, described in the latter section, to hg19 coordinates using UCSC Genome Browser’s liftOver tool. Subsequently, we selected imputed SNPs in the Allan Ancient DNA Resource 1240K panel [44]. These data were then merged with those for the Bimbapes and 1137 individuals from present-day populations from Europe, the Middle East, and North and sub-Saharan African populations [44]. The resulting dataset consisted of 3138 individuals with 141,801 common SNPs. Present-day high-coverage individuals were used to build a PCA using smartpca v.18140 [45], and then ancient and other low-coverage individuals were projected.

## 3. Results

### 3.1. Sample Description

A total of 1054 participants were included in the El Hierro Genome Study, which represents around 9% of the El Hierro population. Of those, 799 had grandparents who were all born on the island, and 416 were unrelated. Table 1 summarizes the sociodemographic data, health habits (smoking), and pathologies presented by all the subjects included in the study (*n* = 1054) and in the subset of unrelated participants (*n* = 416), while Table S1 presents more detailed information. Women were slightly more represented in the study than men (54.0%), and the mean age was 58.0 in the overall sample. Less than half of the participants in each group had completed an elementary education, and less than 20% had completed university studies. From the participants who provided information on smoking, around 60% of both groups declared to have never smoked, and less than 20% declared themselves to be active smokers. The most common diseases were cardiovascular pathologies (Table 1), especially those related to metabolic syndrome, such as high blood pressure and hypercholesterolemia (Table S1). Endocrine pathologies, including diabetes, were also quite common, representing 40.7% of all participants’ conditions. Genitourinary, musculoskeletal, digestive, and neurological pathologies were prevalent, ranging between 20–30% of all conditions. Psychiatric, oncological, respiratory pathologies, and allergies presented a prevalence between 10–20%. Otorhinolaryngological and autoimmune pathologies were the least prevalent (<10%). The largest differences between the two groups of related and unrelated individuals were in the cardiovascular, respiratory, and allergic pathologies (5–8%). The rest of the diseases have similar percentages between groups.

Table 2 summarizes the comparison between the ten most frequent conditions reported in the European Health Interview Survey (EHIS) in Spain (EESE) 2020 [46] and the El Hierro Genome Study. High blood pressure and hypercholesterolemia were the most prevalent conditions in Spain, similar to the El Hierro Genome’s data. However, in El Hierro, these were more common, especially regarding hypercholesterolemia, which was three times higher (50.66% vs. 15.29%). Osteoporosis was the third most prevalent condition in Spain (14.37%), being slightly slower in our sample (12.61%). Unlike the survey carried out in El Hierro, the EESE divided the types of chronic back pain (lumbar or cervical), with a reported prevalence of 11–14%. However, only 2% of the El Hierro participants indicated that they had back pain. Three other conditions were more prevalent in the El Hierro Genome Study than in Spain, including diabetes (3-fold more prevalent; 18.6% vs. 10.0%), anxiety (1.9-fold higher; 11.19% vs. 10%), and allergies (1.8-fold higher). In contrast, varicose veins were six times less prevalent in El Hierro than in Spain. 

### 3.2. Ancestry Inference

**Figure 2:**
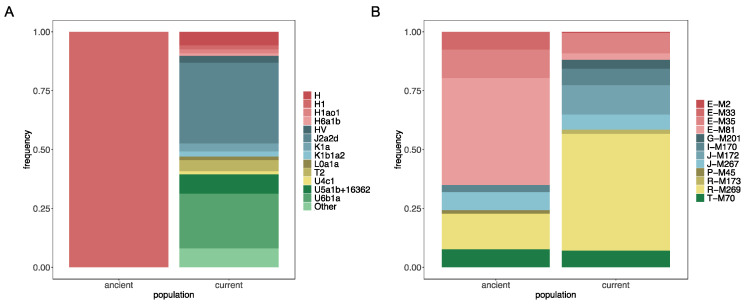
Haplogroup frequencies for mtDNA (**A**) and the Y chromosome (**B**) for both the ancient and modern populations of El Hierro, with the exception of the ancient Y-chromosome frequencies that were calculated for the indigenous people of the whole archipelago.

MtDNA analysis was performed based on 1246 SNPs, producing HaploGrep quality values for haplogroup classification that ranged between 82.2% and 100%. The observed frequencies (Table S2) are similar to those obtained based on a smaller sample from El Hierro obtained by García-Olivares et al. [24]. The haplogroup with the higher frequencies are J2a2d (34.46%) and U6b1a (23.37%) (Figure 2A). These lineages have been previously observed in the indigenous people of the Canary Islands [6,8] and indicate their contribution to the present-day maternal pool in El Hierro. Specifically, J2a2d has been observed in natives from La Gomera and La Palma, while U6b1a has been observed in the indigenous people from La Gomera, Tenerife, and Gran Canaria. It is noteworthy that neither J2a2d nor U6b1a has been found in the ancient population of El Hierro [5,8]. The extreme isolation of El Hierro may have led to the fixation of just one mtDNA lineage, belonging to the H1cf haplogroup [9]. Although identifying H1cf in our dataset is impossible as position 16260 is not included in the Illumina Global Screening array, we were able to classify participants within haplogroup H1 (position 3010), which only accounted for 1.69% of the sample (Supplementary Table S2). It is possible that H1cf is not present in our dataset or it is present at a low frequency as it was not observed by García-Olivares et al. [24] through the analysis of complete mtDNA sequences of 106 individuals. The striking differences between the mtDNA frequencies of the ancient and current populations of El Hierro, and particularly the low frequency of H1 currently, indicate that Bimbapes did not significantly contribute to the current genetic pool [8]. However, the presence of mtDNA lineages frequently observed in the indigenous population suggests the contribution of natives from other islands.

For the chromosome Y (Figure 2B), reliable haplogroup classification was possible for 196 of 197 subjects. Haplogroup assignment qualities ranged from 88.5% to 100%. In the present-day population of El Hierro, the most frequent haplogroup is R-M269 (48.97%), followed by J-M172 (12.75%) (Table S3). The impact of European colonization is evident when comparing the frequencies observed for the Canarian indigenous people with those of the present-day population of El Hierro (Figure 2B). Insufficient information is available from the Y chromosome of the Bimbapes to estimate haplogroup frequencies, so we compared them to frequencies of the indigenous people of the whole archipelago. The haplogroup with the highest frequency in the indigenous people is E-M81 (57.1%) [3,9] a linage that is autochthonous in North Africa and particularly frequent in Western Sahara (76.9%), Morocco (62.1%), and Algeria (82.1%) [47,48]. On the other hand, the most frequent lineage today in El Hierro is R-M269 (Figure 2). Although this haplogroup is also present in North Africa (~5%) and the indigenous people of the Canary Islands (~7.1%) [9,21], it is particularly abundant in populations with European ancestry, reaching a frequency of 60% in the Iberian Peninsula [18]. 

**Figure 3:**
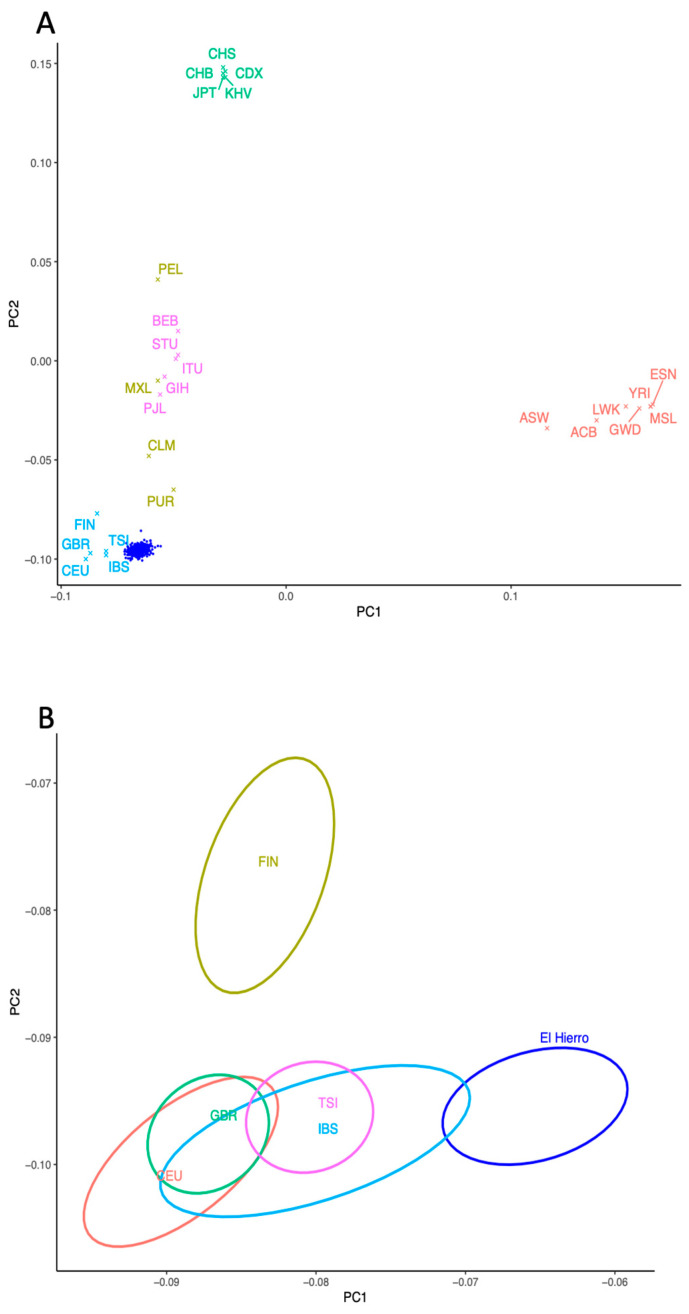
PCA of the El Hierro Genome Study and the 1000 Genomes dataset. (**A**) El Hierro individuals and all super populations from the 1000 Genomes Project (ACB = Afro-Caribbeans in Barbados; ASW = people with African ancestry in southwest USA; ESN = Esan in Nigeria; GWD = Gambians in the Western Division, Mandinka; LWK = Luhyas in Webuye, Kenya; MSL = Mendes in Sierra Leone; YRI = Yorubas in Ibadan, Nigeria; CLM = Colombians in Medellin, Colombia; MXL = people with Mexican ancestry in Los Angeles, California, USA; PEL = Peruvians in Lima, Peru; PUR = Puerto Ricans in Puerto Rico; CDX = Chinese Dai in Xishuangbanna, China; CHB = Han Chinese in Beijing, China; CHS = Han Chinese in South China; JPT = Japanese in Tokyo, Japan; KHV = Kinh people in Ho Chi Minh City, Vietnam; CEU = Utahans with Northern and Western European ancestry; FIN = Finns in Finland; GBR = British people from England and Scotland UK; IBS = Iberian populations in Spain; TSI = Toscani people in Italia; BEB = Bengalis in Bangladesh; GIH = Gujarati Indians in Houston, Texas, USA; ITU = Indian Telugus in the UK; PJL = Punjabis in Lahore, Pakistan; STU = Sri Lankan Tamils in the UK). (**B**) Detail of the clustering of El Hierro with European populations (CEU = Utahans with Northern and Western European ancestry; FIN = Finns in Finland; GBR = British people from England and Scotland UK; IBS = Iberian populations in Spain; TSI = Toscani people in Italia).

**Figure 4:**
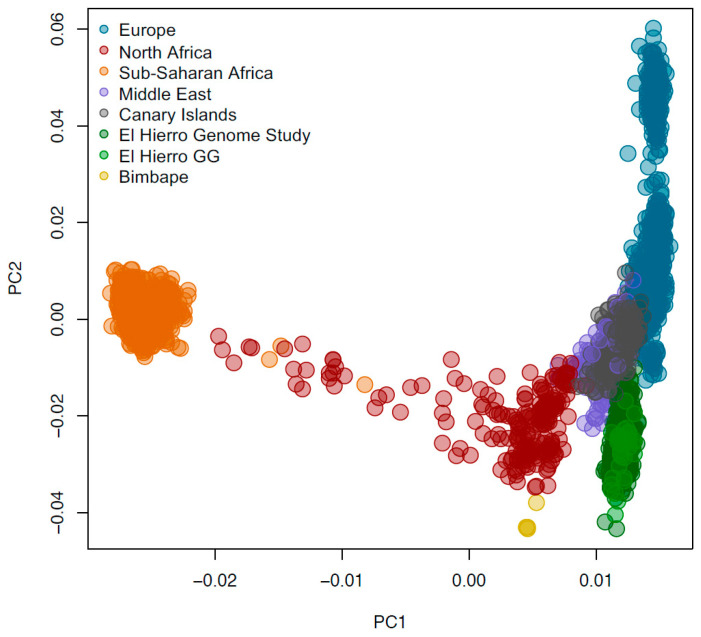
PCAs of El Hierro compared to populations from Europe, the Middle East, North Africa, and sub-Saharan Africa, as well as the present-day Canary Islands individuals and ancient individuals from El Hierro. Code: The El Hierro Genome Study refers to individuals analyzed in this study, and El Hierro GG refers to individuals from El Hierro previously genotyped by Guillén-Guío et al.

The PCA of autosomal SNPs showed that when compared to the 1000 Genomes data, the El Hierro samples cluster adjacent to European populations (particularly IBS) (Figure 3A). However, due to its demographic history, El Hierro is shifted toward the African populations in PC1 (Figure 3B). The El Hierro population was then compared to a dataset comprising European, Middle Eastern, North African, and sub-Saharan populations, as well as previously genotyped present-day individuals from the Canary Islands [23] and four low-coverage ancient genomes from El Hierro [9]. In this PCA, PC1 separates sub-Saharan and Eurasian populations, and PC2 differentiates El Hierro (including the indigenous people and present-day individuals) and North Africa from Europe (Figure 4), with the remaining modern populations from the Canary Islands placed in the middle. The placing of El Hierro apart from the other Canarian individuals can be due to the higher sample size of El Hierro compared with the remaining populations, and the possible differentiation of this island due to isolation.

### 3.3. Runs of Homozygosity

**Figure 5:**
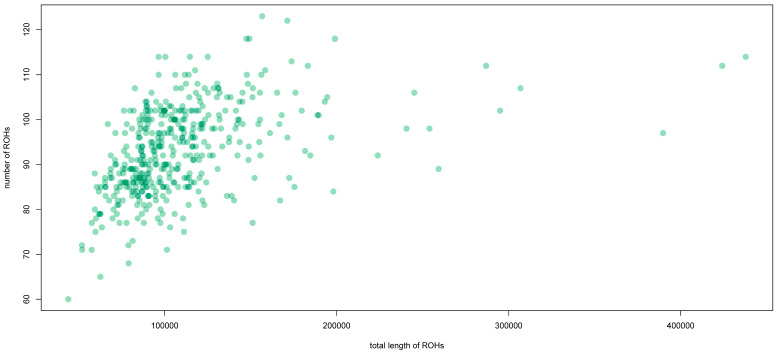
Number of ROHs compared to their total length for the population of El Hierro.

To investigate parental relatedness and consanguinity in the population of El Hierro, we performed an estimation of the presence of runs of homozygosity (ROHs) in the present-day population of the island. ROHs occur when identical haplotypes are inherited from each progenitor, giving information on population history [49], and they may also be involved in the individual susceptibility to diseases [50]. Most individuals from El Hierro had less than 150 Mb of their genome in ROHs (87.8%). Eleven individuals had more than 200 Mb of their genome in ROHs, while three of them had around 400 Mb (Figure 5). These ROH signatures are similar to what has been observed in the ancient peoples of the island [9]. Serrano et al. [9] determined that all Bimbape individuals had more than ~150 Mb of their genome in ROHs, with one of them exceeding ~500 Mb. Although results obtained for present-day and ancient populations cannot be compared because they were inferred using different genotyping methods, it suggests that similar effects of isolation may have affected the island since ancient times.

## 4. Discussion

In this manuscript, we present the characteristics of the El Hierro Genome Study, which includes 1054 participants representing 9% of the whole island population. We confirmed the genetic admixture origin of the El Hierro population, which clustered adjacent to the Iberian population but with a shift toward the African population. Moreover, when analyzing the ROH, we observed signs of genetic isolation. We showed that some pathologies had a high prevalence in this population, including cardiovascular, endocrine, genitourinary, musculoskeletal, digestive, and neurological disorders. 

Studies comparing disease prevalence across Spain have pointed out that cardiovascular risk factors, such as hypertension, dyslipidemia, and diabetes, show a higher prevalence in the Canary Islands than in other Spanish territories [51]. In the case of diabetes, the strikingly high prevalence observed is not described in other European territories [52]. These data agree with our results, where the prevalence of the three mentioned conditions presents at a significantly higher rate. Taking into account that cardiovascular risk factors are the main cause of death in Spain [53], our sample may have special relevance for the study of genetic and environmental factors related to these conditions. Likewise, future research can benefit from the conditions with a higher prevalence in our sample, such as allergies, which have already been documented to have a high prevalence in the islands [54], or other conditions to be explored within mental health, such as anxiety. Additionally, the possible protective factors related to the idiosyncrasy of the island of El Hierro can be advantageously studied in diseases that have a lower prevalence compared with national ones, such as osteoporosis and, notably, varicose veins and chronic back pain. 

Like other regions with a colonial history, the current population of the Canary Islands is the result of gene flow from different geographical areas. More specifically, this process involved the admixture of the Canarian indigenous people with colonists from Europe (mainly the Iberian Peninsula), as well as enslaved people brought from North Africa and sub-Saharan Africa [12]. The same components have been observed for the island of El Hierro. In this case, mtDNA results suggest that, rather than being contributed by Bimbapes, the indigenous component observed in the current population could result from admixture with natives from other islands when contact between islands increased after the European conquest [8,9]. Another aspect that characterizes El Hierro is the effect of insularity on its population, both in ancient [9] and present times [23]. Although this is more pronounced in the indigenous people, we observe that some present-day individuals have an important portion of their genomes in ROHs that exceed 400 Mb. This result indicates that the same effects that isolation and insularity had on the indigenous people before the conquest also affected the El Hierro population in historical and recent times.

As personalized medicine advances, the need to catalog the specific genetic variations in each population increases, taking special relevance in genetically isolated populations, such as El Hierro. In this scenario, understanding the genetic structure of the El Hierro population drives us to an ideal scenario where the characteristics of an isolated population allow the identification of unique variants that could be more easily related to the risk of suffering from a disease [28]. In fact, this population offers a unique opportunity to better understand the influence of ROH on complex diseases by means of homozygosity mapping, allowing the identification of genomic regions with recessive effects on complex traits [49]. This could allow the confirmation of already known genetic risk factors identified by genome-wide association studies in other populations and also reveal novel genomic regions related to complex traits not previously described [49]. Additionally, due to the admixture composition of the El Hierro population, derived from European and African components, admixture mapping analyses of different complex traits can be performed, which have been shown to be able to reveal additional loci that do not achieve stringent GWAS thresholds in other populations [55,56,57], including Canary Islanders [58].

The El Hierro Genome Study possesses several strengths and limitations that warrant discussion. Among its strengths, it is noteworthy as the first study to sample a substantial proportion of the population of El Hierro (9%), coupled with comprehensive health status data records. Therefore, this study is open for collaborations to map different complex traits. Additionally, as mentioned before, the isolated but admixed component of the population allows different types of analyses to be applied for disease mapping, potentially revealing variants with lower frequency in other populations that could have achieved a higher frequency in El Hierro. Moreover, all the participants have similar environmental exposures related to rural living, minimizing the effects of environmental factors. Furthermore, the presence of a family history of all the diseases investigated was recorded for each participant, allowing for the future selection of controls for different traits that have no history of the specific disease under study. However, certain limitations need to be acknowledged in the El Hierro Genome Study. Firstly, while some quantitative traits related to blood tests were recorded in the patients (such as glucose levels, cholesterol levels, and blood cell counts), other measurements, such as height, weight, or lung function, were not included. Moreover, sampling was restricted to blood in EDTA tubes, which only allowed genomic or epigenomic analyses to be performed in the El Hierro population, but not transcriptomic or microbiome studies. Although the sample size represents a large proportion of the total population, it can still be considered limited for performing GWAS analyses.

## 5. Conclusions

In summary, this study confirmed the admixed origin of the El Hierro population as an admixture of European and North African components, which had a differential contribution in the maternal and Y-chromosome lineages. Additionally, signs of isolation were identified. Therefore, these findings underscore that the El Hierro Genome Study presents a valuable opportunity to investigate the genetic underpinnings of various diseases within an isolated understudied population.

## Figures and Tables

**Table 1:** Summary of sociodemographic information and disease prevalence in the El Hierro Genome Study participants.

Variable	Whole Population *n* = 1054	Unrelated Individuals *n* = 416
Age (years)	58.0 (46–72)	59.6 (50–63)
Sex (female)	570 (54.0)	219 (52.6)
Ancestors (all grandparents from El Hierro)	799 (75.8)	416 (100)
Education level (*n* = 1034/*n* = 409)		
Elementary only	427 (41.3)	189 (46.24)
Higher than elementary	388 (37.5)	143 (35.04)
University completion	201 (19.4)	68 (16.6)
Other	18 (1.7)	8 (2.0)
Smoking (*n* = 1044/*n* = 415)		
Never smoker	600 (57.5)	246 (59.3)
Former smoker	253 (24.2)	101 (24.3)
Current smoker	191 (18.3)	67 (16.1)
Respiratory diseases	153 (14.5)	34 (8.2)
Allergy	196 (18.6)	53 (12.7)
Cardiovascular pathologies	703 (66.7)	310 (74.5)
Endocrine pathologies	429 (40.7)	173 (41.6)
Autoimmune pathologies	50 (4.7)	17 (4.1)
Musculoskeletal pathologies	245 (23.2)	111 (26.7)
Genitourinary pathologies	253 (24.0)	90 (21.6)
ORL pathologies	71 (6.7)	26 (6.3)
Digestive system pathology	296 (28.1)	117 (28.1)
Oncological pathologies	137 (13.0)	69 (16.6)
Neurological pathologies	296 (28.1)	112 (26.9)
Mental disorders	159 (15.1)	58 (13.9)
Other pathologies not specified	395 (37.5)	169 (40.6)

The descriptive variables are represented by the median (interquartile range) for continuous variables and the count (proportion) for categorical variables.

**Table 2:** Comparison between the ten most prevalent conditions in Spain according to the EESE and the participants from the El Hierro Genome Study.

Variable	EESE Spain *n* = 22,072	Whole Population El Hierro Genome Study *n* = 1054
High blood pressure	19.3%	38.89%
Hypercholesterolemia	15.29%	50.66%
Osteoporosis	14.37%	12.61%
Chronic back pain (lumbar)	13.69%	1.89%
Chronic back pain (cervical)	11.33%	
Allergy	10.0%	18.59%
Varicose veins	7.54%	1.23%
Diabetes	7.5%	20.68%
Migraines/Cephalalgia	6.75%	8.34%
Anxiety	5.84%	11.19%

## Data Availability

All the data supporting the findings and conclusions of this study are reported and available in the main text and/or Supplementary Materials of this article. For clinical and other omics data generated within the El Hierro Genome Study, these data will be made available upon specific request subject to the requestor obtaining ethical, research, data access, and collaboration approvals from the study management board. Requests can be sent to martagpuga@gmail.com, mdelpino@ull.edu.es, and patrick.sullivan@ki.se.

## Supplementary Materials

The following supporting information can be downloaded at: https://www.mdpi.com/article/10.3390/jpm14060626/s1, Table S1 (Sociodemographic information and disease prevalence in the El Hierro Genome Study participants), Table S2 (Absolute and relative mtDNA haplogroup frequencies for El Hierro population) and Table S3 (Absolute and relative Y-chromosome haplogroup frequencies for El Hierro population).
