# Supplementary material for: El Hierro Genome Study: A Genomic and Health Study in an Isolated Canary Island Population
Source: J Pers Med. 2024 Jun 12;14(6):626. doi: 10.3390/jpm14060626 (PMC11204744; doi:10.3390/jpm14060626)
Supplement: Supplementary file 1 [file jpm-14-00626-s001.zip › jpm-3007904-supplementary.pdf]

Table S1. Sociodemographic information and disease prevalence in the El Hierro Genome Study participants.

Variable	Whole population n=1,054	Unrelated individuals n=416
**Age (years)**	58.0 (46-72)	59.6 (50-63)
Age >40	933	397
**Sex (female)**	570 (54.0)	219 (52.6)
**Ancestors (all grandparents from El Hierro)**	799 (75.8)	416 (100)
**Education level (n = 1034/ n = 409)**		
Elementary only	427 (41.3)	189 (46.2)
Higher than elementary	388 (37.5)	143 (35.0)
University completion	201 (19.4)	68 (16.6)
Other	18 (1.7)	8 (2.0)
**Smoking (n= 1044 / n = 415)**		
Never smoker	600 (57.5)	246 (59.3)
Former smoker	253 (24.2)	101 (24.3)
Current smoker	191 (18.3)	67 (16.1)
**Respiratory diseases**	**153 (14.5)**	**34 (8.2)**
Apnea	3 (0.3)	1 (0.2)
Asthma	82 (7.8)	5 (1.2)
Chronic obstructive pulmonary disease	47 (4.5)	18 (4.3)
Pulmonary hypertension	0	0
Other pulmonary disease	42 (4.0)	15 (3.6)
**Allergy**	**196 (18.6)**	**53 (12.7)**
Food allergy	17 (1.6)	6 (1.4)
Drug allergy	83 (7.9)	25 (6.0)
Atopic conjunctivitis	9 (0.9)	2 (0.5)
Atopy	9 (0.9)	0
Allergic rhinitis	54 (5.1)	10 (2.4)
Other allergies	68 (6.5)	19 (4.6)
**Cardiovascular pathologies**	**703 (66.7)**	**310 (74.5)**
Angina pectoris	4 (0.4)	3 (0.7)
Hypercholesterolemia	534 (50.7)	245 (58.9)
Arterial hypertension	410 (38.9)	182 (43.8)
Ictus	15 (1.4)	5 (1.2)
Myocardial infarction	20 (1.9)	10 (2.4)
Varicose veins	13 (1.2)	8 (1.9)
Other cardiovascular pathology	154 (14.6)	62 (14.9)
**Endocrine pathologies**	**429 (40.7)**	**173 (41.6)**
Diabetes	218 (20.7)	102 (24.5)
Hyperthyroidism	15 (1.4)	4 (1.0)
Hypothyroidism	115 (10.9)	50 (12.0)
Other endocrine pathology	205 (19.4)	70 (16.8)

**Autoimmune pathologies**	**50 (4.7)**	**17 (4.1)**
Arthritis	14 (1.3)	7 (1.7)
Crohn's disease	2 (0.2)	0
Sclerosis	0	0
Lupus erythematosus	3 (0.3)	3 (0.7)
Vitiligo	7 (0.7)	1 (0.2)
Other autoimmune pathology	28 (2.7)	8 (1.9)
**Musculoskeletal pathologies**	**245 (23.2)**	**111 (26.7)**
Osteoporosis	133 (12.6)	67 (16.1)
Chronic back pain	20 (1.9)	9 (2.2)
Osteoporosis	35 (3.3)	18 (4.3)
Other musculoskeletal pathology	96 (9.1)	36 (8.7)
**Genitourinary pathologies**	**253 (24.0)**	**90 (21.6)**
Prostate hyperplasia	60 (5.7)	31 (7.5)
Renal insufficiency	32 (3.0)	15 (3.6)
Uterine myoma	25 (2.4)	10 (2.4)
Polycystic ovaries	27 (2.6)	4 (1.0)
Other genitourinary pathology	151 (14.3)	44 (10.6)
**ORL pathologies**	**71 (6.7)**	**26 (6.3)**
Tonsillitis	15 (1.4)	1 (0.2)
Otitis	4 (0.4)	0
Nasal polyps	6 (0.6)	2 (0.5)
Sinusitis	6 (0.6)	2 (0.5)
Vegetations	10 (0.9)	3 (0.7)
Other ORL pathology	39 (3.7)	18 (4.3)
**Digestive system pathologies**	**296 (28.1)**	**117 (28.1)**
Chronic constipation/diarrhea	8 (0.8)	3 (0.7)
Hemorrhoids	13 (1.2)	2 (0.5)
Liver failure	3 (0.3)	0
Gastroesophageal reflux	46 (4.4)	20 (4.8)
Intestinal polyposis	48 (4.5)	21 (5.0)
Gastroduodenal ulcer	21 (2.0)	11 (2.6)
Other digestive system pathology	232 (22.0)	90 (21.6)
**Oncological pathologies**	**137 (13.0)**	**69 (16.6)**
Colon cancer	10 (0.9)	5 (1.2)
Breast cancer	21 (2.0)	11 (2.6)
Prostate cancer	24 (2.3)	8 (1.9)
Lung cancer	4 (0.4)	2 (0.5)
Bladder cancer	5 (0.5)	1 (0.2)
Leukemia	1 (0.09)	0
Lymphoma	0	0
Melanoma	3 (0.3)	2 (0.5)
Myeloma	0	0
Benign tumor	24 (2.3)	13 (3.1)
Other oncological pathology	55 (5.2)	31 (7.5)

**Neurological pathologies**	**296 (28.1)**	**112 (26.9)**
Migraine/Cephalalgia	88 (8.3)	34 (8.2)
Epilepsy	12 (1.1)	6 (1.4)
Neurodegenerative disorders	22 (2.0)	10 (2.4)
Other neurological pathology	89 (8.4)	41 (9.9)
**Mental disorders**	**159 (15.1)**	**58 (13.9)**
Anxiety	118 (11.2)	46 (11.1)
Depression	112 (10.6)	40 (9.6)
Other psychiatric conditions	165 (15.5)	14 (3.4)
**Other pathologies not specified**	**395 (37.5)**	**169 (40.6)**

Descriptives are represented by the median (interquartile range) for continuous variables and the count (proportion) for categorical variables.

Table S2. Absolute and relative mtDNA haplogroup frequencies for El Hierro population.

MtDNA haplogroup	Absolute frequency	Relative frequency
H	24	5.78%
H1	7	1.69%
H1e	1	0.24%
H1v	1	0.24%
H1aa1	3	0.72%
H1ao1	7	1.69%
H3af	1	0.24%
H6a1b	4	0.96%
H7a	1	0.24%
H13a	1	0.24%
H13a2b3	2	0.48%
H22	1	0.24%
H29	1	0.24%
HV	12	2.89%
J2a2d	143	34.46%
K1a	14	3.37%
K1b1a2	9	2.17%
L0a1a	6	1.45%
L2a1f2	1	0.24%
L3d1b3	1	0.24%
L3x2b	1	0.24%
T1	1	0.24%
T2	20	4.82%
T2c1d1	1	0.24%
U3a1	2	0.48%
U4a2a	2	0.48%
U4a3	1	0.24%
U4c1	5	1.20%
U5a'b	3	0.72%
U5a1b	1	0.24%

U5a1b+16362	34	8.19%
U5b2a	1	0.24%
U6b1a	97	23.37%
U6c1	1	0.24%
W	2	0.48%
X2b	2	0.48%
X3	1	0.24%

Table S3. Absolute and relative Y-chromosome haplogroup frequencies for El Hierro population. It is important to note that a portion of the Y-chromosome data available from the indigenous people was obtained by amplifying specific Y-chromosome markers by PCR. For that reason, it was needed to group some more-derived lineages obtained in the present-day population into major haplogroups to be able to compare both datasets. The correspondence for the haplogroup classification based on the PCR-based method and the recalculated frequencies are shown in the last two columns.

**Haplogroup**	**Y-chromosome marker**	**Absolute frequency**	**Relative frequency**	**Correspondence with Y-chromosome markers genotyped in the indigenous people**	**Relative frequency for comparison**
E1b1a1a1c1a1	P252	1	0,51%	E-M2 (E1b1a)	0,51%
E1b1b	M215	17	8,67%	E-M35* (E1b1b1), including E-M78 (E1b1b1a1) and E-M34 (E1b1b1b2a1a)	8,67%
E1b1b1b1a	M81	6	3,06%	E-M81 (E1b1b1b1a)	3,06%
G2a2b2a1a1a	Z2022	2	1,02%	G-M201 (G)	4,08%
G2a2b2b1a	F935	6	3,06%		
I1	S66	8	4,08%	I-M170 (I)	7,14%
I1a1b	L22	4	2,04%		
I1a3	Z63	1	0,51%		
I2a1a	L158	1	0,51%		
J1a	Z2215	13	6,63%	J-M267 (J1)	6,63%
J2a	M410	8	4,08%	J-M172 (J2)	12,75%
J2a1b	M67	14	7,14%		
J2b2a	L283	3	1,53%		
R1a1a1b	S224	1	0,51%	R-M173 (R1), including E-M17 (R1a1a)	1,53%
R1a1a1b1	S339	2	1,02%		
R1b1a2a	L23	3	1,53%	R-M269 (R1b1a1b)	48,97%
R1b1a2a1a	L151	56	28,57%		
R1b1a2a1a1c1a	S498	15	7,65%		
R1b1a2a1a2a1a1	S181	4	2,04%		
R1b1a2a1a2b	S28	13	6,63%		
R1b1a2a1a2c	L21	2	1,02%		
R1b1a2a1a2c1f2	Z2534	3	1,53%		
T1a1a	CTS2611	11	5,61%	T-M70 (T1a)	6,63%
T1a2b	L446	2	1,02%		
